# The response of salivary proinflammatory biomarkers to tooth extraction in individuals with type II diabetes mellitus

**DOI:** 10.1186/s12903-024-04006-1

**Published:** 2024-02-19

**Authors:** Yousuf Ibrahim Al Shehhi, Noha M. Elemam, Mohammed Amjed Alsaegh

**Affiliations:** 1 Department of Oral and Craniofacial Health Sciences, College of Dental Medicine, University of Sharjah, Sharjah, UAE; 2 Department of Clinical Sciences, College of Medicine, University of Sharjah, Sharjah, UAE; 3 Research Institute for Medical and Health Sciences, University of Sharjah, Sharjah, UAE

**Keywords:** Proinflammatory, Salivary cytokines, Tooth extraction, TNF-α, IL-6, IL-1β, IFN-γ

## Abstract

**Purpose:**

This study investigated the levels of salivary proinflammatory cytokines in the saliva of patients living with type II diabetes mellitus (DM) compared to those in healthy individuals three times: before tooth extraction and at 2 hours and 2 days after tooth extraction.

**Methods:**

The study included 27 participants. Among them, 20 (*n* = 20; 74%) had type II DM, and seven (*n* = 7; 26%) were healthy control subjects. Saliva samples were collected at three time intervals: before tooth extraction and 2 hours and 2 days after tooth extraction. The salivary biomarkers were investigated using a Luminex multiplex assay. These salivary biomarkers included tumor necrosis factor-alpha (TNF-α), interleukin 6 (IL-6), interleukin 1-beta (IL-1β), and interferon-gamma (IFN-γ).

**Results:**

At baseline, patients with type II DM had significantly lower levels of IL-1β (*P* = 0.016). Moreover, 2 hours after extraction, patients with type II DM had significantly lower levels of IL-1β and TNF-α than did healthy control subjects (*P* = 0.046 and *P* = 0.020, respectively). In addition, 2 days after tooth extraction, the DM group had significantly greater IL-6 levels (*P* = 0.010) than the control group.

**Conclusions:**

In patients with type II DM, salivary proinflammatory biomarker levels are generally comparable or lower than those in healthy control subjects. Proinflammatory cytokines manifest differently in patients with type II DM after tooth extraction than in normal healthy individuals. There is generally a delayed early response of salivary proinflammatory markers in patients living with type II DM who undergo tooth extraction.

## Introduction

Diabetes mellitus (DM) is a metabolic disease characterized by hyperglycemia caused by defects in insulin secretion, insulin action, or both. Type II DM is more prevalent than type I DM, and the overall burden will likely continue to increase in the future. In the absence of urgent and adequate action, there are predictions that 578 million people will have diabetes in 2030, and 700 million people will have diabetes in 2045 [1].

The process of bone healing in the extraction socket is delayed in patients with diabetes [2]. Physiological, inflammatory, immune, endocrine, and neural factors contribute to the recovery period of extraction sockets in patients with diabetes. Dysregulation of growth factors and cytokines contributes to longstanding wound healing in diabetes patients [3].

Hyperglycemia produces glycation end-products (AGEs). The interaction between AGEs and their receptors leads to intracellular signaling and the release of proinflammatory molecules [4]. Consequently, patients with DM are thought to experience a greater inflammatory response and produce more inflammation-related cytokines [5]. Several proinflammatory biomarkers, which could be useful diagnostic tools for wound healing in the oral cavity, have been detected in the saliva of patients with diabetes. A complex process is involved in the healing of extraction sockets, including the reconstruction of soft and hard tissues damaged during the procedure. Numerous cytokines are involved in the regulation of this process [3].

After tooth extraction surgery, endocrine, immunological, and hematological changes occur, leading to inflammation, followed by proliferative and remodeling processes. Multiple molecules are released during the healing process, where they cause a local inflammatory immune response [6].

These molecules have been described as having an important role in bone healing. The cytokines regulate cellular movement and infiltration to facilitate repair [7]. Currently, there is no consensus regarding the clinical difference in the healing of tooth extraction sockets between individuals with and without DM.

A growing body of evidence indicates that saliva can be used as a diagnostic tool with rapid advancements in nanotechnology and molecular diagnostics [8]. Traditionally, biomarkers have been measured by serum sampling because blood contains a high concentration of molecular components. The difference between blood analysis and salivary analysis is that blood analysis examines protein-bound, serum-circulating compounds, whereas salivary analysis examines biologically active molecules at the cellular level, suggesting that salivary analysis provides a more accurate picture [9]. A salivary inflammatory biomarker is a biomolecule, such as a protein or derivative, that can be measured to evaluate inflammation in the mouth [10]. Additionally, salivary biomarkers are promising sources for monitoring oral and systemic pathologies due to their ease of collection, noninvasive nature, and accuracy of diagnosis [6]. However, the use of saliva as a diagnostic tool faces a number of challenges. First, saliva contains extremely low concentrations of molecular components. Second, studies on salivary biomarkers that are specific to diseases are rare [11]. A previous study showed a positive correlation between salivary inflammatory biomarkers and serum HbA1c levels in patients with type II DM [12]. However, this increase in inflammatory cytokines such as IL-6 and TNF-α in saliva may be attributed not only to DM but also to periodontitis. Thus, additional studies are clearly needed to determine whether there is indeed a unique salivary biomarker profile associated with DM [5].

In this study, proinflammatory salivary biomarkers were measured at baseline and at two postsurgical intervals in patients with type II DM and healthy control subjects. The involved salivary biomarkers include TNF-α, interleukin 1-beta (IL-1β), interleukin 6 (IL-6), and interferon-gamma (IFN-γ).

## Methods

### Study design and participants

Twenty-seven patients were recruited from the University Dental Hospital of Sharjah between 1/10/2021 and 21/9/2022. Among them, 20 patients had type II DM, and seven were healthy control subjects. The study sample consisted of patients whose teeth were scheduled for extraction in accordance with the oral surgery protocol. The study included participants aged older than 18 years who had previously been diagnosed with type II DM for at least 1 year. Patients with type II DM had to have a recent glycated hemoglobin level (HbA1c) below 7.5% according to the glycated hemoglobin test and a random glucose level below 180 mg/dL according to glucometer-based measurements. In the DM group, all patients were well controlled and had no diabetes-related systemic disease. None of the patients in the control group had a history of systemic disease. The indications for extraction in both groups were nonrestorable necrotic teeth with no periodontal or apical lesions. Patients who smoked or used any type of tobacco were excluded, as were patients with immunological diseases or immunosuppressive treatments, antibiotic or anti-inflammatory therapy, acute infections or abscesses originating from the dental system, pregnant or breastfeeding women, those who needed prophylactic antibiotics before dental treatments, and those with any other systemic conditions or diseases that would influence current or previous inflammatory responses.

The sample size was calculated using the statistical software G*Power (ver. 3.1.9.7 software; University of Düsseldorf, Germany). Based on the information provided by Gutiérrez-Corrales et al. [10], we used an effect size of 0.5, an 80% power, and a 5% error, and 21 patients were required. Therefore, this study included a convenience sample of 27 patients. The purpose of the study was explained to the participants before an informed consent form was signed and obtained from each of them. The study was conducted in accordance with the guidelines of the Declaration of Helsinki. The University of Sharjah Human Research and Ethics Committee approved this study (REC-21-06-01-03-S).

### Saliva sample collection

A 5-ml sample of unstimulated whole saliva was collected from each patient three times: before extraction, 2 hours after extraction, and 2 days after extraction. Before sample collection, patients were asked not to eat or drink for 1 hour. The participants were asked to gently rinse their mouth with tap water for 2 minutes and then spit into the collection tube over a period of 5 minutes. To prevent disturbing blood clots, patients were instructed to allow passive saliva to flow into the collection tube after extraction. After being stored in an ice-cold box, all the samples were frozen at − 80 °C until analysis.

### Measurement of salivary biomarkers

The TNF-α, IL-1β, IL-6, and IFN-γ concentrations in saliva were evaluated using a Luminex multiplex assay. All the collected samples were brought to room temperature and centrifuged at 4000 rcf for 15 minutes at 4 °C. The supernatant was transferred to a clean microcentrifuge tube, 75 µl of which was diluted with 75µl of calibrant diluent from the assay kit. The concentrations of the different cytokines were measured using a Human Magnetic Pre-Mixed Analyte Luminex Assay (R&D Systems, UK). The standard cocktail was prepared according to the manufacturer’s instructions, followed by 3-fold serial dilution to obtain 7 standards. After the preparation of all the standards and samples, 50 µl of the respective standards and samples were added to the respective wells of the 96-well plate, along with 50 µl of the premixed microparticle cocktail. The samples were incubated for 2 hours at room temperature on an orbital shaker at 800 rpm. Then, the wash buffer was diluted 25 times in distilled water and utilized for the washing steps performed using a Bio-Plex Washing Station (Bio-Rad Laboratories, USA). Next, 50 µl of the diluted biotin-antibody cocktail was added to the plate and left for 1 hour on an orbital shaker. Washing was repeated, followed by the addition of 50 µl of diluted streptavidin-PE for 30 minutes on an orbital shaker. Finally, the microparticles were resuspended in 100 µl of wash buffer, and fluorescence was detected using the Bio-Plex-200 system (Bio-Rad Laboratories, USA). Analysis of the data was performed using Bio-Plex Manager software (Bio-Rad Laboratories, USA). The standard curve was calculated using the 5-parameter logistic curve method. The respective fluorescence readings of the samples were converted to concentrations in pg/ml, which were subsequently multiplied by 2 to obtain the original concentrations of the samples.

### Statistical analysis

The data were collected, tabulated, and analyzed statistically. SPSS ver. 28 (IBM Corporation, Armonk, NY, USA) was used for the statistical analysis. Quantitative variables are presented as the mean, standard deviation, coefficient of variation, range, and standard error. Two-tailed t tests were conducted for paired samples and independent samples. A significance level was set at *P* < 0.05.

## Results

### Participants

The acute salivary proinflammatory cytokine response at baseline and after simple tooth extraction was investigated in 27 patients. Table 1 shows the demographic information of the participants.

**Table 1:** Demographic data of the study participants

Participants (27)		
Total	Diabetes mellitus group	Control group
27	20	7
Gender		
Total	Diabetes mellitus group	Control group
Male =20	Male = 16	Male = 4
Female = 7	Female = 5	Female = 2
Mean age		
Total	Diabetes mellitus group	Control group
51.67 ± 13.26	57.95 ± 7.48	33.71 ± 8.77

### Salivary biomarker concentrations

#### TNF-α

The mean concentrations of TNF-α in the DM group were 29.42 pg/ml, 19.83 pg/ml, and 35.46 pg/ml before extraction, 2 hours after extraction and 2 days after extraction, respectively. Nevertheless, in the nondiabetes group, the concentrations were 39.57 pg/ml, 47.82 pg/ml, and 37.52 pg/ml before extraction, 2 hours after extraction and 2 days after extraction, respectively. At 2 hours after extraction, the TNF-α concentration in the DM group was significantly lower than that in the nondiabetes group (*P* = 0.02) (Table 2).

**Table 2:** Comparison of salivary TNF-α, IL-6, IL-1β, and IFN-γ levels between type-2 diabetic patients and control individuals at baseline, 2 hours after tooth extraction and 48 hours after tooth extraction

No.	Marker	group	number	Baseline levels		2 hours after extraction		48 hours after extraction	
				Mean ± SD pg/ml	*P* value	Mean ± SD pg/ml	*P* value	Mean ± SD pg/ml	*P* value
1	TNF-α alpha	Diabetes mellitus	20	29.42 ± 20.903	.335	19.83 ± 11.761	.020*	35.46 ± 31.435	.075
		Control	7	39.57 ± 16.387		47.82 ± 38.108		37.52 ± 14.424	
2	IL-6	Diabetes mellitus	20	101.96 ± 172.645	.740	239.49 ± 412.353	.678	416.76 ± 618.496	.010**
		Control	7	91.73 ± 133.547		375.62 ± 431.612		141.09 ± 100.244	
1.3	IL-1β	Diabetes mellitus	20	1481.91 ± 1450.406	.016*	991.29 ± 683.816	.046*	2166.88 ± 2126.603	.261
		Control	7	4418.61 ± 5004.515		1804.47 ± 1834.959		2460.50 ± 1285.304	
4	IFN-γ	Diabetes mellitus	20	65.17 ± 36.501	.135	55.71 ± 30.514	.463	70.30 ± 43.867	.084
		Control	7	88.66 ± 26.147		78.20 ± 27.253		73.62 ± 29.802	

* = Significant

** = Highly significant

**Fig. 1:**
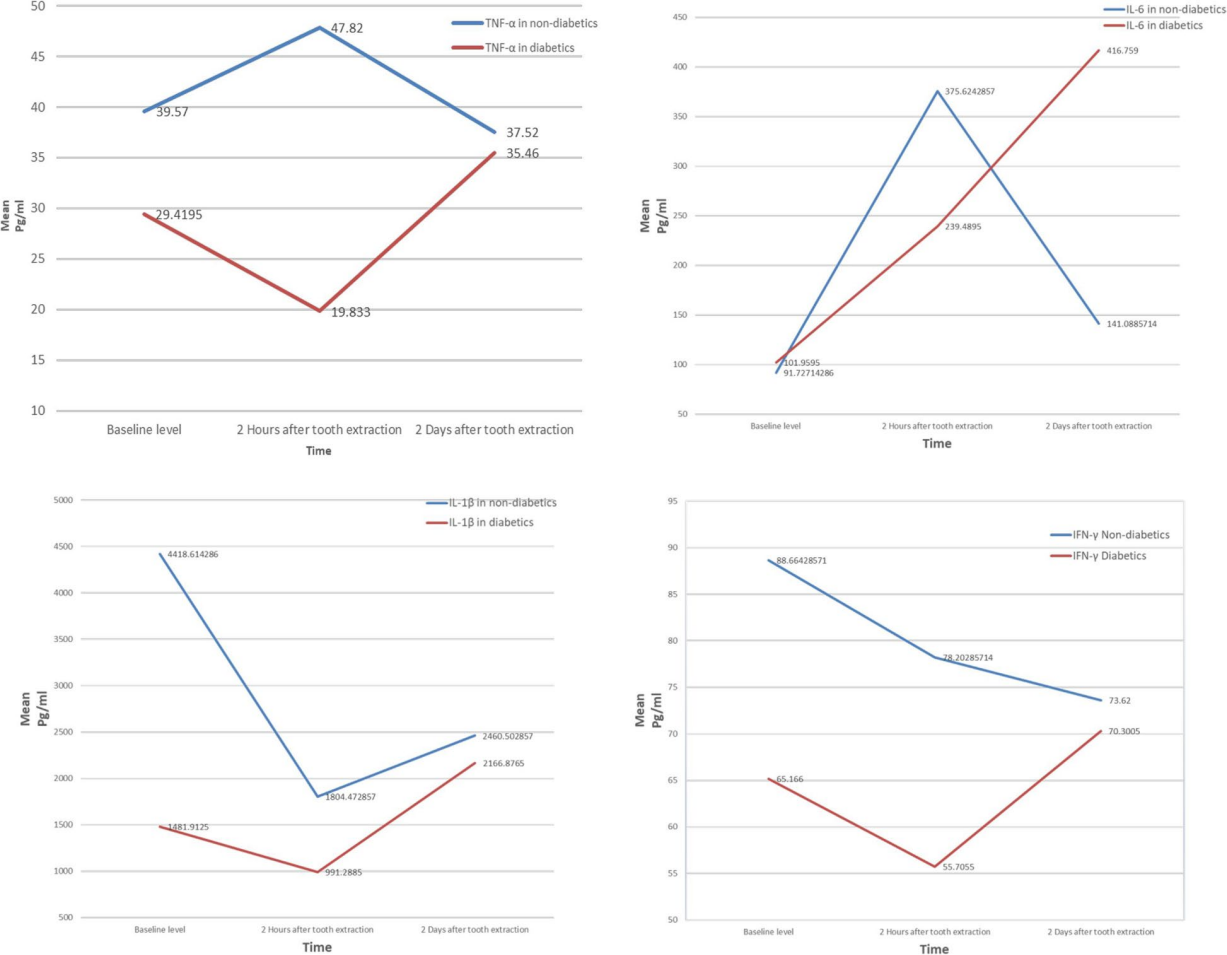
Line graphs of TNF-α, IL-6, IL-1β, and IFN-γ at baseline, 2 hours after tooth extraction and 48 hours after tooth extraction for individuals with type 2 diabetes and control individuals

The level of TNF-α decreased in the DM group 2 hours after extraction and then returned to increase 2 days after extraction. In contrast, the control group showed increases in TNF-α 2 hours after extraction, followed by a decrease 2 days later. Nevertheless, during all studied time intervals, diabetes patients had lower TNF-α levels than healthy control subjects did (Fig. 1).

#### IL-6

The mean concentrations of IL-6 in the DM group were 101.96 pg/ml, 239.49 pg/ml, and 416.76 pg/ml before extraction, 2 hours after extraction and 2 days after extraction, respectively. In the nondiabetes group, the concentrations were 91.73 pg/ml, 375.62 pg/ml, and 141.09 pg/ml before extraction, 2 hours after extraction and 2 days after extraction, respectively. IL-6 levels in the DM group were significantly greater than those in the nondiabetes group at 2 days post-extraction (*P* = 0.010) (Table 2).

IL-6 levels in the studied and control groups were approximately equal at baseline. Following extraction, the IL-6 concentration in both groups increased, with the control group showing a greater increase. The level of IL-6 in the DM group continued to increase 2 days later, whereas it was reduced in the control group (Fig. 1).

#### IL-1β

The mean concentrations of IL-1β in the DM group were 1481.91 pg/ml, 991.29 pg/ml, and 2166.88 pg/ml before extraction, 2 hours after extraction and 2 days after extraction, respectively. In the nondiabetes group, the concentrations were 4418.61 pg/ml, 1804.47 pg/ml, and 2460.50 pg/ml before extraction, 2 hours after extraction and 2 days after extraction, respectively. A significantly lower concentration of IL-1β was observed in the DM group than in the nondiabetes group at baseline (*P* = 0.016) and at 2 hours after extraction (*P* = 0.046) (Table 2). In both patients with type II DM and healthy control subjects, salivary IL-1β levels decreased 2 hours after extraction and then increased after 2 days (Fig. 1).

#### IFN-γ

The mean concentrations of IFN-γ in the DM group were 65.17 pg/ml, 55.71 pg/ml, and 70.30 pg/ml before extraction, 2 hours after extraction and 2 days after extraction, respectively. In the nondiabetes group, the concentrations were 88.66 pg/ml, 78.20 pg/ml, and 73.62 pg/ml before extraction, 2 hours after extraction and 2 days after extraction, respectively. The concentration of the IFN-γ biomarker in saliva was greater in the control group than in the DM group. However, these differences were not statistically significant at the three time points (*P* > 0.05) (Table 2).

In both the DM and control groups, IFN-γ levels decreased from the baseline level to 2 hours after tooth extraction. Although the IFN-γ level increased in the DM group to a level greater than baseline on the second postextraction day, it continued to decrease in the control group (Fig. 1).

## Discussion

Extraction of teeth is one of the most common oral surgeries. During wound healing in the extraction socket, inflammation occurs when inflammatory cells influx into the wound to clear dead cells, debris, and potential pathogens, allowing endothelial cells, fibroblasts, and keratinocytes to migrate, proliferate, and differentiate to repair the wound. However, uncontrolled DM has a negative effect on wound healing [13]. While several studies have indicated impaired wound healing in patients with DM, questions still remain as to what kind of local and systemic factors contribute to prolonged wound healing [14]. In fact, regulating wound healing is critical, where inappropriate proinflammatory signaling can result in wounds taking longer to heal and being more susceptible to infection [15]. There are specific cytokines involved in each stage of wound healing, so abnormal or imbalanced local cytokine concentrations may explain the impaired healing process [16]. Moreover, it has been suggested that the immune response and inflammation are related to alveolar bone resorption observed after tooth extraction in a porcine model [17].

Proinflammatory cytokines are among the first factors that are released in response to wounds, and they regulate the functions of immune cells. As part of wound healing, proinflammatory cytokines, such as TNF, IL-1, IL-6, and IL-17, participate in the inflammatory phase through the activation of downstream cascades [18]. Immune responses are, however, a double-edged sword in wound healing. It is detrimental to produce excessive amounts of proinflammatory cytokines. Nevertheless, moderate immune responses can promote wound healing, which helps to prevent infection and facilitate the healing process [19].

In concordance with our results, a previous study concluded that most growth factors that promote wound healing are deficient in diabetic patients, resulting in delayed and impaired healing of oral mucosal wounds [14]. At the base line, we found that the salivary TNF-α and IL-1β concentrations were lower than those of the healthy control subjects, and this difference was statistically significant for IL-1β. This finding was in agreement with another study that found lower levels of TNF-α, IL-4, INF-γ, RANTES and IL-7 in the DM group compared to the control group, indicating that type II DM modulates the local expression of molecules involved in anti-inflammatory and healing processes [20]. In contrast, a previous study revealed that salivary proinflammatory biomarker levels are greater in patients with type II DM than in healthy control subjects [12].

In the present study, TNF-α and IL-6 levels in saliva increased in healthy control subjects 2 hours after extraction, whereas IL-1β and IFN-γ levels decreased. However, patients with type II DM experienced similar increases in IL-6 but not TNF-α on Day 2 after tooth extraction. There was a decrease in all of the control groups after 2 hours, except for IL-1β, which increased. Conversely, patients with type II DM showed an increase in the levels of all the examined proinflammatory markers on Day 2. In light of the above discussion, patients with type II DM appear to have delayed responses and a tendency to have increased levels of inflammatory cytokines over time. It has been proposed that during early wound healing, there is no inflammatory storm in patients with DM, but there is excessive and persistent inflammation during long-term nonhealing wounds. During acute wound patterns, impaired peripheral neuropathy and unstabilized mast cells blunt the ability to generate an acute proinflammatory response, whereas chronic wounds may cause persistent inflammation due to overflowing M1 macrophages [21].

TNF-α is an important factor in wound healing since it stimulates angiogenesis and cellular processes such as proliferation, differentiation, growth, and the immune response. Furthermore, TNF-α is important for necrosis and apoptosis, which results in the removal of damaged cells and foreign bodies from wound sites [22]. In the present study, we observed that patients with type II DM had a lower baseline level of TNF-α than did healthy control subjects. Moreover, TNF-α was significantly lower in patients with type II DM following tooth extraction than in healthy control subjects. Biological processes such as angiogenesis, differentiation, growth, and immunity might be altered by a reduction in TNF-α levels at baseline and 2 hours after extraction in patients with type II DM, resulting in delayed wound healing after extraction. A previous study proposed that there should be an optimal concentration of TNF-α in fracture callus, which enhances early healing. Consequently, excessive or insufficient TNF secretion may have a detrimental effect on the healing process [23]. The altered proinflammatory cytokine responses observed in our study may be linked to the previously observed slower healing of wounds after tooth extraction in patients with DM than in healthy control subjects [24].

This effect of TNF-α could delay bone healing in patients with DM following tooth extraction. Previous studies have shown contradictory results in the comparison of TNF-α levels between patients with DM and healthy individuals. Several previous studies have shown that elevated salivary baseline levels of TNF-α are significantly associated with type II DM [6, 12, 25, 26]. Another previous study revealed no statistically significant difference in TNF-α between diabetic patients and control individuals [27]. However, Srinivasan et al. reported that the baseline level of salivary TNF-α was significantly lower in patients with DM than in healthy control subjects [28]. This discrepancy may be due to differences in the sample type, duration of the diabetes disease process, methodology and specimen used, oral hygiene, or presence of associated comorbidities. Our results revealed that there was no statistically significant difference in the baseline level of TNF-α between the diabetes group and the control group. Moreover, we detected an increase followed by a decrease in TNF-α in healthy control subjects at 2 hours and 2 days after tooth extraction. These findings are in agreement with those of a previous study in which TNF-α synthesis increased following wound injury, peaked on Day 1, and then decreased to a basal level on Day 2 [29]. Moreover, a previous study demonstrated a reduction in TNF-α levels in patients with DM after dental extraction [6]. One can propose the use of TNF-α inhibitors shortly after tooth extraction in normal individuals to avoid this peak in TNF-α, which could be harmful to the healing socket.

IL-6 plays a key role in modulating inflammatory and reparative processes. It is essential for the activation, differentiation, and proliferation of endothelial cells, leukocytes, fibroblasts, and keratinocytes [30]. In response to inflammation, macrophages secrete IL-6, which is involved in the recruitment and apoptosis of leukocytes, as well as the activation of T cells [5]. One can propose targeting IL-6 as part of a combination treatment with growth factors, as IL-6 plays a significant role in both the inflammation and proliferative phases of wound healing. Therefore, inflammation may be controlled without compromising wound healing. It is imperative that the inflammatory response be initiated and resolved at the right time for the wound to heal successfully. When the IL-6 signaling pathway is impaired, wound healing may be delayed [30].

We found almost comparable baseline levels of IL-6 in patients with type II DM and control subjects. A prior study revealed that salivary IL-6 levels were greater in patients with type II DM than in control individuals, but the difference was not statistically significant [12]. However, other reports have shown that salivary levels of IL-6 are significantly greater in individuals with type II DM than in healthy individuals [28, 31]. Our results demonstrated that, 2 hours after tooth extraction, the IL-6 level was greater in healthy individuals than in patients with type II DM, but the difference was not significant. Similarly, a previous study revealed that, compared with that in nondiabetic wounds, IL-6 expression in diabetic wounds was significantly lower 6 hours after injury.15 Nevertheless, we found that patients with type II DM had a statistically significant increase in IL-6 at 48 hours after tooth extraction compared to that in the control group. In accordance with our study, a previous study revealed that the levels of the proinflammatory marker IL-18 levels post-extraction were higher in patients with DM than in healthy individuals, suggesting that these individuals have a greater inflammatory load [6].

In the present study, IL-1β levels were significantly lower in the DM group than in the nondiabetic group both at baseline and 2 hours after tooth extraction. This reduction, however, was not significant at 48 hours postoperatively. It has been suggested that IL-1 acts as a chemotactic factor for fibroblasts. It appears that IL-1 plays an important role in tissue remodeling and repair in many ways, similar to TNF-α [32].

Contrary to our findings in acute diabetic wounds, there is evidence that IL-1β levels are increased in chronic diabetic wounds such as human diabetic foot ulcers, where it inhibits the healing process [33]. Moreover, blocking IL-1β in type II DM patients was associated with decreased IL-6 and TNF-α and improved wound healing [34]. It is well documented that the levels of IL-1β are elevated in chronic wounds. However, there is little understanding of its level and effect on acute wound healing in patients with DM.

IFNs are produced by cells in response to stimulation provided by certain bacteria, viruses, mitogens, and antigens. There are three major types of IFNs: alpha (a), beta (b), and gamma (g) (IFN-γ). The immune IFN IFN-γ is produced in response to specific antigens or mitogens. Stimulated lymphocytes are the primary source of IFN-γ. IFNs perform antiviral, antiproliferative, and immunomodulatory functions. Chemotaxis and fibroblast proliferation, as well as collagen production, are inhibited by IFNs. Overall, its effects on fibroblasts appear to be antifibrotic [32]. Postoperatively, we found a continuous decrease in IFN-γ levels in healthy individuals. Moreover, patients with type II DM had elevated levels of IFN-γ 2 days after surgery. However, how IFN-γ contributes to wound healing remains controversial and poorly understood. Compared with those in wild-type mice, IFN-γ-deficient mice exhibited reduced myofibroblast differentiation, wound closure, and wound breaking strength [35].

For the first time, this study identified differences in the acute response of proinflammatory cytokines to oral injury caused by tooth extraction between patients with type II DM and healthy control subjects. There was a significant difference between patients with type II DM and healthy control subjects in terms of the pattern of the acute response to proinflammatory cytokines. These findings suggest that patterns of wound healing in patients with type II DM may be influenced by these cytokines. In patients with type II DM, wound healing may be influenced by targeting and optimizing these cytokines. This study is limited by the wide range of ages and locations of tooth extraction sites, as well as the limited number of time intervals studied. In this study, only the difference in the inflammatory cytokine response between patients with DM and those without DM was observed. It is recommended that further research be conducted to identify the specific pathway involved in these differences as well as their impact on wound healing. Furthermore, additional information regarding patients’ BMI and medications is necessary, and further investigations are required to examine the immunological background of patients and to compare such data within groups of patients with different HbA1c levels.

## Conclusion

In patients with type II DM, salivary proinflammatory biomarker levels are generally comparable or lower than those in healthy control subjects. Compared with those in healthy individuals, proinflammatory cytokines in patients with type II DM differ after tooth extraction. There is generally a delayed early response of salivary proinflammatory markers in patients with type II DM who have had tooth extractions. The findings of this study pave the way for further investigations into whether these differences affect postoperative wound healing and complications following tooth extraction in patients living with type II DM.

## Data Availability

The datasets generated and analyzed during the current study are available in the Mendeley Data Repository (https://data.mendeley.com/datasets/2pf6p9gdxk/1).

## Abbreviations

TNF-α: Tumor necrosis factor-alpha
IL-6: Interleukin 6
IL-1β: Interleukin 1-beta
IFN-γ: Interferon-gamma
DM: Diabetes mellitus
AGEs: Hyperglycemia produces glycation end-products
HbA1c: Glycated hemoglobin level
SPSS: Statistical package for the social sciences.
